# Hydrothermally synthesized cobalt selenide graphene nanocomposite as a sensitive probe for electrochemical profiling of dopamine and uric acid

**DOI:** 10.1039/d5ra08825a

**Published:** 2026-07-02

**Authors:** Munira Khalid, Musarrat Younas, Abid Ali, Arif Nazir, Amel Y. Ahmed, Imene Bayach, Warda Mansur, Murat Kaleli, Salih Akyürekli

**Affiliations:** a Department of Chemistry, Women University of Azad Jammu & Kashmir Bagh 12500 Pakistan; b Department of Chemistry, The University of Lahore 1-Km Defence Road Lahore 54590 Pakistan abid.ali@chem.uol.edu.pk; c Department of Chemistry, Faculty of Science, King Faisal University Al Ahsa 31982 Saudi Arabia; d Suleyman Demirel University, Innovative Technologies Application and Research Center 32260 West Campus Çünür Isparta Turkey; e Department of Chemistry, Faculty of Science, Universiti Malaya Kuala Lumpur 50603 Malaysia

## Abstract

Electrochemical sensing is an affordable and efficient method for detecting a wide range of biologically, medically, and environmentally important substances. Carbon-based materials, such as graphene and carbon nanotubes, are commonly employed in the fabrication of electrochemical sensors due to their unique structures and advantageous properties including high electrocatalytic activity, minimal surface fouling, low cost, biocompatibility, and efficient electron transfer kinetics. This study proposes the hydrothermal synthesis of graphene based cobalt selenide (CoSe@GO) nanocomposites for electrochemical sensing of uric acid and dopamine. The as-fabricated electrode's structure and morphology were examined using scanning electron microscopy (SEM) coupled with energy-dispersive X-ray spectroscopy (EDS). X-rays diffraction (XRD) was employed to examine the crystalline structure and phase composition of the microelectrode material. While electrochemical performance was evaluated using various electrochemical methods, including cyclic voltammetry and chronoamperometry. The fabricated CoSe-GO@GCE sensor provides a good linear range of up to 1.6 mM and 2.0 mM with a low detection limit of 1.5 mM and 0.03 mM for dopamine and uric acid, respectively. By developing these advanced nanocomposites, this research seeks to enhance sensitivity, selectivity, and detection limits for uric acid and dopamine, contributing to improved biomedical diagnostics and monitoring.

## Abstract 2

Electrochemical sensing is an affordable and efficient method for detecting a wide range of biologically, medically, and environmentally important substances.

## Graphical abstract

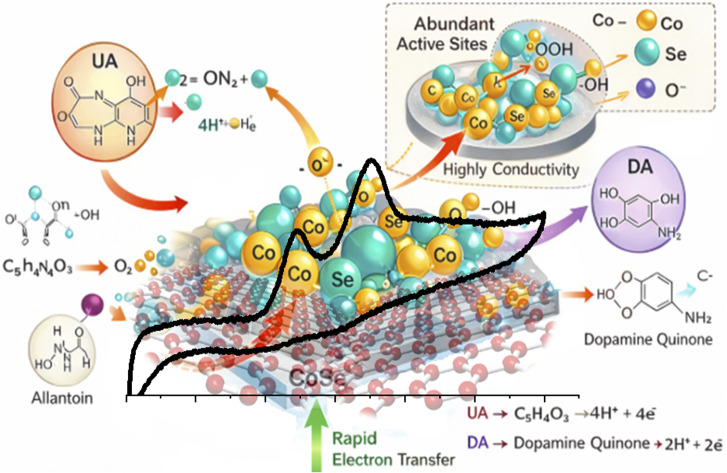

## 1 Introduction

Electrochemical sensors represent a cost-effective and simple analytical technique that delivers high sensitivity, excellent detection limit, repeatability and ease of miniaturization.^1,2^ These sensors have a powerful analytical paradigm, synergistically uniting the high sensitivity of electroanalytical techniques with the exquisite selectivity of a biological recognition element.^3,4^ The operational principle involves the specific interaction, either catalytic or affinity based, between the biorecognition element and its target analyte. This event is then transduced into a measurable electrical signal, the magnitude of which is directly proportional to the analyte's concentration.^5^ The success and versatility of this technology are underscored by its widespread commercialization and routine application in diverse fields, including clinical diagnostics, environmental monitoring, industrial process control, and agriculture. Among various electrode materials, graphene (GR) has gained considerable attention for analyzing substances relevant to food quality, pharmaceuticals, and environmental monitoring, owing to its unique physicochemical properties.^6^ Compared to carbon nanotube (CNT) based electrodes, GO based electrodes exhibit superior electrocatalytic activity and improved conductivity at the macroscopic scale.^7^ The high surface area, low over potential, and rapid electron transfer kinetics of GO based sensors contribute to low limits of detection (LOD) and swift response times.^8^ Materials such as graphene, graphene oxide (GO), and reduced graphene oxide (rGO) are highly promising for fabricating nanocomposites and electrochemical sensors.^9^ Graphene is especially attractive due to its outstanding thermal conductivity, mechanical strength, and chemical stability.^10^ These characteristics, along with its high electron mobility, flexibility, and robustness, are critical for bioanalytical sensing applications. Structurally, GO consists of a single atomic layer of sp^2^-hybridized carbon atoms arranged in a two-dimensional honeycomb lattice. Its low electronic noise combined with excellent electrical, optical, thermal, mechanical and electrochemical properties, makes it an ideal material for electrochemical sensing (ECS).^2^ Because of its remarkable and intriguing properties, such as the quantum Hall effect, high Young's modulus, superior electrical conductivity, charge carrier mobility, extensive surface area, superior transparency, and robust chemical stability and strong mechanical strength, graphene has garnered a lot of attention lately.^11^ These exceptional characteristics make graphene-based materials intriguing for use in extensive variety of industrial and technical domains. Over the past decade, substantial progress has been made in the synthesis and application of graphene and graphene-based nanocomposites, facilitating the development of electrochemical sensors for diverse analytes in fields such as medical diagnostics, environmental monitoring, bioanalysis, and security.^12^

Cobalt oxide (Co), a transition metal oxide has found extensive usage in electrochemical applications because of its favorable chemistry, simplicity of tailoring its shape, ease of production, and abundance in nature.^13^ The synergistic combination of graphene oxide (GO) and cobalt oxide (Co) has attracted a lot of attention because of its unique properties and potential applications.^14^ It is a well-known electrocatalyst with excellent redox properties and high catalytic activity and has been used widely in energy storage devices, sensors, and catalysts; however, its practical applications are often limited by its low electrical conductivity and poor stability.^15^ By combining GO and Co, researchers have created GO–Co composites that exhibit enhanced properties compared to their individual components. The integration of Co nanoparticles onto the GO surface can greatly improve the composite's electrical conductivity, stability, and catalytic activity.^16^

The distinctive optical, physical, and chemical characteristics of selenium nanoparticles (SeNPs) with various morphologies have opened up significant opportunities for the advancement of biosensors in biomedical applications.^17^ Their outstanding biocompatibility, electrical conductivity, catalytic efficiency, high surface-to-volume ratio, and dense structural features have made SeNPs widely utilized in the development of electrochemical biosensors, offering superior analytical performance compared to traditional sensor designs.^18^ Among many nanomaterials explored for biosensing, they have attracted considerable attention for their ability to effectively link biological recognition events with signal transduction. This makes them highly promising for the creation of innovative biosensing platforms aimed at detecting, quantifying, and analyzing a wide array of biologically important analytes.^19^

Graphene oxide combined with cobalt selenium (CoSe-GO) composite have become attractive options for biosensing applications because of their special set of characteristics.^16^ Because of its exceptional electrical conductivity, graphene oxide (GO) makes electrochemical biosensors more effective in transferring electrons. Enzymes, antibodies, DNA, and other biomolecules may be immobilized on GO's high surface area, which boosts the biosensor's sensitivity.^20^ When GO and CoSe are combined in a composite material, they may have synergistic effects that improve biosensor performance even more.^21^ The biologically active molecules such as uric acid (UA) and dopamine (DA) coexist in the human body and play essential roles. Dopamine is a member of the catecholamine family and has garnered a lot of interest because of its important role in message transmission, cognitive function, and clinical diagnosis in both the central and peripheral nervous systems. A high DA level can cause a number of illnesses, including addiction, Alzheimer's disease, and Parkinson's disease.^22^ The primary metabolite of purine metabolism that is generated in our blood and serum is UA. UA typically ranges between 300 and 500 µM in serum and between 1400 and 4400 µM in urine. Hyperuricemia, gout, arthritis, Lesch–Nyhan syndrome, and kidney lesions are among the conditions that can be brought on by an excessive amount of UA.^23^ Thus, it is crucial to precisely identify them in studies and provide a diagnosis in a clinic.^24^

In this work, a cobalt selenide-based graphene nanocomposite was synthesized *via* a facile hydrothermal process, and the material was utilized as a catalyst for the electrochemical detection of uric acid and dopamine. Can be converted into passive voice. The shape and structure of CoSe@GO nanocomposite were examined using XRD, SEM and EDS. Before the validation of the efficacity of the developed sensor, the sensitive layer was characterized electrochemically. As fabricated electrode (CoSe@GO) exhibited the efficient performance with a low detection limit of 1.5 mM and 0.03 mM and linear range of up to 1.6 mM and 2.0 mM for the dopamine and uric acid, respectively.

## 2 Experimental

### 2.1 Materials

The chemical reagents used such as cobalt chloride (CoCl_2_·6H_2_O), selenium powder uric acid, dopamine and potassium hydroxide (KOH) were obtained from Sigma-Aldrich. Graphene was synthesized using a modified Hummer's method. All solvents employed in the experiments were of analytical grade and used as received, without any additional purification. Deionized water was used for the preparation of all solution.

### 2.2 Preparation of CoSe@GO nanohybrids and electrode fabrication

The CoSe@GO nanohybrids were made using a hydrothermal process. Stoichiometric amounts of selenium (Se) powder and cobalt chloride hexahydrate (CoCl_2_·6H_2_O) were taken in a 1 : 1 molar ratio and dissolved in 50 mL of deionized water. CoSe@GO nanohybrids were synthesized through the ultrasonic dispersion of graphene oxide (0.02 g), which had been prepared *via* a modified Hummers' method.^25^ Hydrazine Hydrate was then added dropwise under constant stirring. This addition served to reduce the graphene oxide, enhancing its graphitization, and simultaneously reduced Co^2+^ ions to form cobalt selenide nanoparticles were deposited onto graphene sheets, and the resulting suspension was transferred into a Teflon-lined stainless-steel autoclave. The mixture was then heated at 120 °C for 12 hours. After naturally cooling to room temperature, the resulting precipitate was thoroughly washed with distilled water and dried in a vacuum oven at 50 °C for 12 hours, yielding the CoSe@GO nanohybrids. This synthesis procedure was used to prepare CoSe@GO nanohybrids with different mass ratios of CoSe to graphene: 1 : 0.25, 1 : 0.50, and 1 : 0.75. For comparison purposes, pure CoSe nanoparticles were also synthesized under identical conditions, except without the inclusion of graphene oxide.^26^

**Scheme 1:**
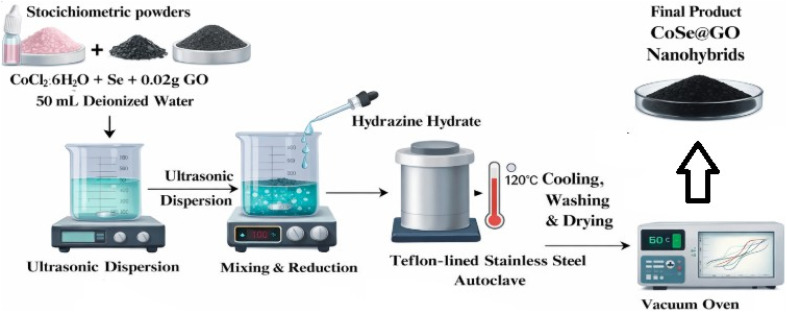
Schematic illustration for the synthesis and electrode fabrication protocol of CoSe@GO.

Electrochemical tests, including cyclic voltammetry and chronoamperometry, were performed using a Gamry Reference 3000 Potentiostat/Galvanostat/ZRA in a conventional three-electrode configuration. Platinum wire served as the counter electrode, Ag/AgCl as the reference electrode, for electrode fabrication, a simple drop-casting method was employed using a slurry. Nafion was employed as a binder in the aqueous mixture and ethanol as a dispersion medium in which the as synthesized nanocomposite sample was accurately weighed and placed into an Eppendorf tube. 20 µL of distilled water was added, followed by 1–2 drops of Nafion binder. The resulting solution was then sonicated for 10 minutes to ensure thorough dispersion of the nano composite. The prepared nanocomposite slurry was carefully applied to the surface of the glassy carbon electrode (GCE) and allowed to dry under ambient conditions (Scheme 1).

The novelty of probe synthesis in this work lies in one-pot hydrothermal co-reduction strategy that enables the *in situ* growth of cobalt selenide nanoparticles directly on graphene sheets. Unlike previously reported CoSe-graphene systems prepared by post-mixing or sequential assembly, this method ensures strong interfacial coupling between CoSe and graphene within the same assembly (one pot), leading to efficient electron transport and suppressed nanoparticle agglomeration. The simultaneous reduction of GO and Co^2+^ ions create a defect rich, highly conductive hybrid network, which is crucial for enhancing electrochemical response. Moreover, the composition-controlled CoSe@GO ratios allow optimization of catalytic activity and conductivity, enabling effective electrochemical discrimination of dopamine and uric acid-an aspect not previously demonstrated in CoSe-based sensing platforms.

## 3 Results and discussion

### 3.1 Surface topography

**Fig. 1:**
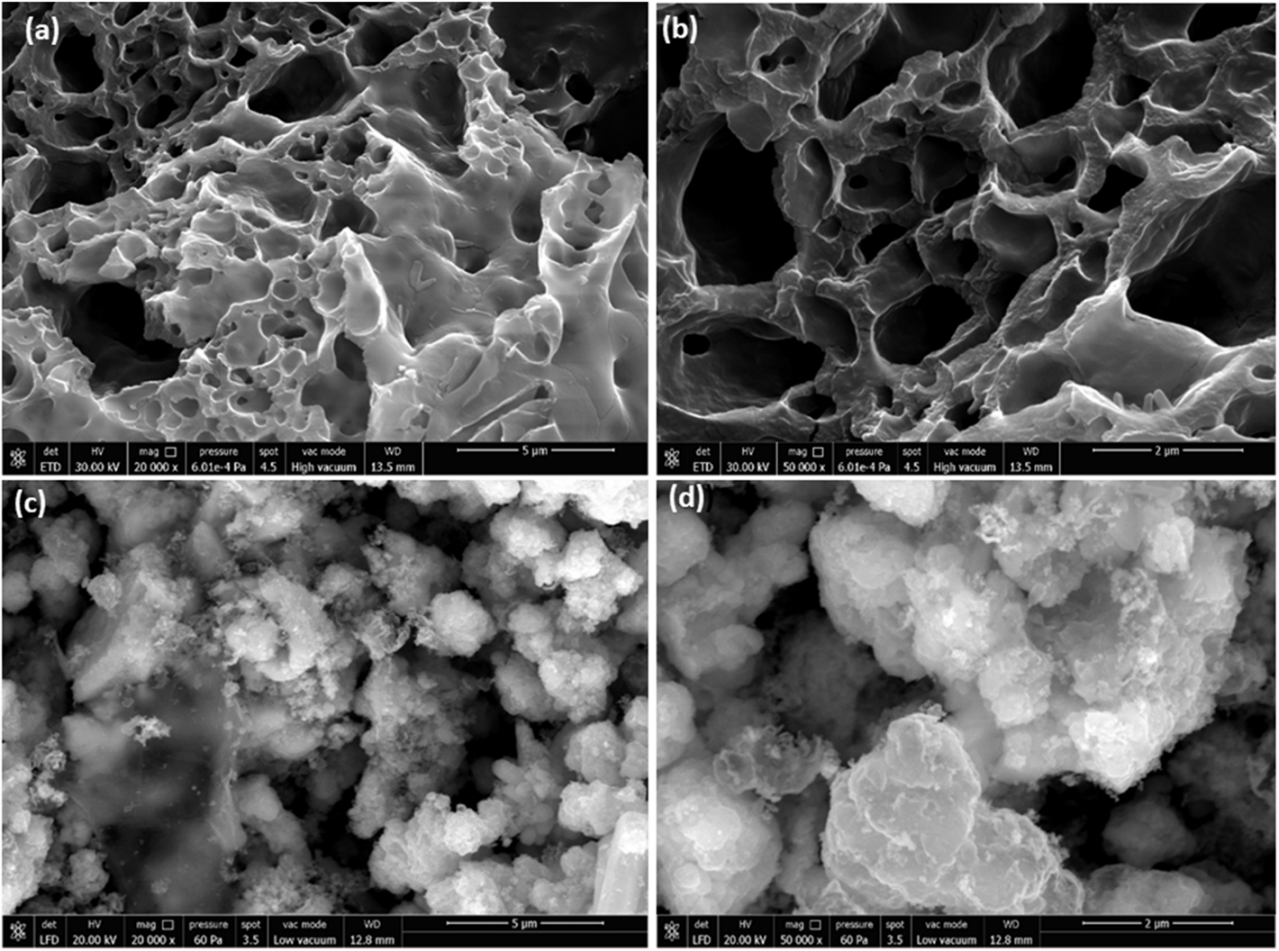
SEM images for bare GO (a) 5 µm and (b) 2 µm magnification and CoSe@GO at (c) 5 µm and (d) 2 µm.

Scanning Electron Microscopy (SEM) was used for high-resolution surface imaging. Fig. 1(a) and (b) shows the SEM micrograph of bare graphene oxide at a 5 µm and 2 µm which reveals a distinctly hollowed and porous morphology. This observation indicates the presence of significant spaces and cavities within the GO material, suggesting a complex, non-solid architecture composed of interconnected layers. The intricate network of these hollows directly contributes to a substantial accessible surface area.^27^ This porous nature, analogous to a microscopic sponge, implies that the GO is not simply a flat sheet but possesses a three-dimensional structure with numerous internal spaces available for interaction. Consequently, the observed hollowed and porous features highlight the potential for enhanced surface interactions, a critical factor for applications exploiting GO's reactivity. Fig. 1(c) and (d) reveals the surface morphology of CoSe@GO, a composite material of cobalt selenide (CoSe) supported on graphene oxide (GO). At a 5 µm magnification (image c), the SEM reveals an agglomerated morphology of the CoSe@GO composite, where distinct CoSe nanoparticles or clusters are seen dispersed and attached to the GO substrate. The GO likely provides a matrix, preventing extensive aggregation of the CoSe. The texture appears rough and granular, indicative of the presence of the CoSe component on the typically flaky GO structure. This image suggests a successful integration of CoSe onto the GO substrate, potentially leading to enhanced properties due to the synergistic effects of the two components, such as increased surface area and improved electrochemical performance. Based on the provided SEM image at a 2 µm scale in Fig. 1(d), the CoSe@GO composite reveals a more detailed surface structure compared to the 5 µm image. At this higher magnification, the image shows a highly agglomerated structure with a rough and granular texture. The CoSe nanoparticles appear more distinct and seem to be densely packed and distributed across the GO sheets. The GO support is less apparent at this scale, with the CoSe dominating the observed morphology. The image indicates a close interaction between the CoSe and GO, with the nanoparticles likely anchored to the GO surface, preventing excessive aggregation. This intimate contact suggests a synergistic effect between the two components, which would enhance the material's properties, such as increased surface area and improved electrochemical performance. The high density of CoSe on the GO support is evident, highlighting the composite's potential for applications where a large active surface area is crucial.^14^

**Fig. 2:**
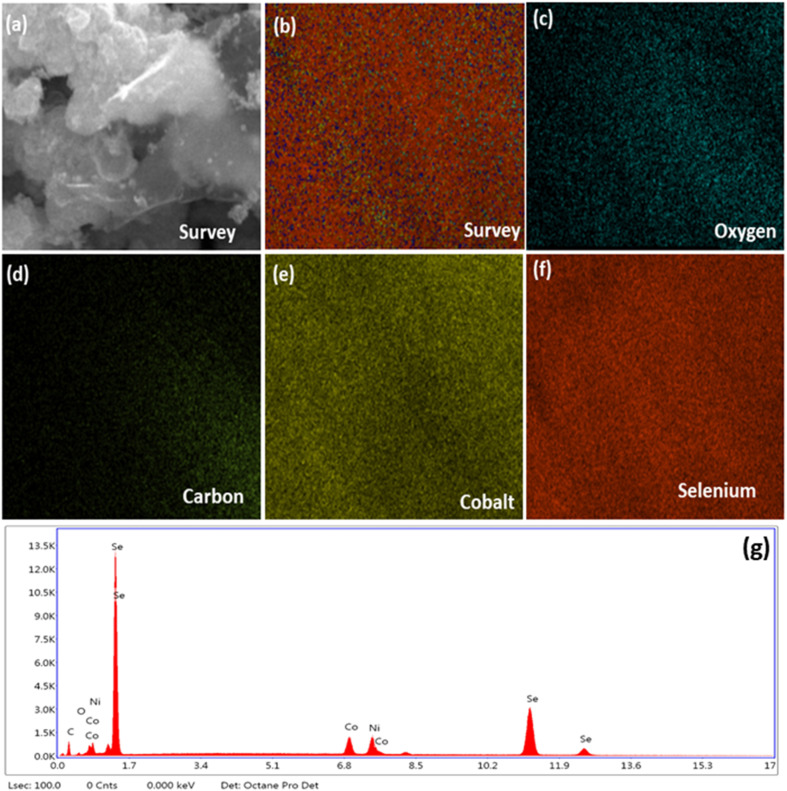
Energy-dispersive X-ray spectroscopy analysis for the CoSe@GO with (a and b) survey analysis and corresponding elemental mapping for (c) oxygen (d) carbon (e) cobalt (f) selenium and (g) the elemental distribution spectra.

Fig. 2 displays an energy-dispersive X-ray spectroscopy (EDX) spectrum for the CoSe@GO composite material identifying the constituent elements and their relative abundance within the sample. EDS is a surface-sensitive technique coupled with SEM was used to provide elemental analysis of the imaged area. The EDS analysis, encompassing elemental mapping, provides compelling evidence for the successful formation of the CoSe@GO composite. The elemental mapping spatially visualizes these elements. The widespread distribution of red (selenium) and green (cobalt) indicates that cobalt selenide is present throughout the analyzed area. These signals appear anchored onto a background of black (carbon), representing the GO sheets. The presence of blue (oxygen) is consistent with the oxygen-containing functional groups on the GO. The relatively uniform distribution of all four colors across the map suggests a homogenous composite material at the microscale, implying that the CoSe nanoparticles or clusters are well-dispersed on the GO support. The strong intensity of the cobalt and selenium signals, relative to carbon and oxygen, hints at a significant amount of cobalt selenide being incorporated into the composite structure. This combined EDX data confirms the intended elemental composition and provides insights into the homogeneity of the CoSe@GO material.

The elemental composition of the fabricated nanocomposite (CoSe@GO) was also characterized through elemental distribution spectra in EDX (Fig. 2(g)). From the EDX spectrum, the occurrence of Co and Se peaks verifies the successful integration of CoSe while the intensity of the C signal can be associated with the entire framework of graphene oxide. In addition, there is also a considerable intensity of the oxygen peak that can be ascribed to the functional groups present on the surface of GO. Nickel as impurities also appeared in the spectra which might be associated with the carbon tapes having trace Ni.

### 3.2 XRD analysis of CoSe@GO composite

**Fig. 3:**
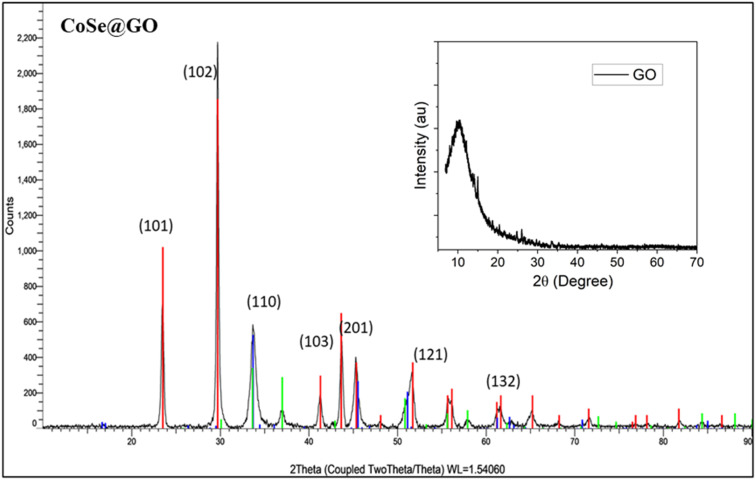
XRD pattern of CoSe@GO composite and GO (inset).

The crystalline structure of the synthesized CoSe@GO composite was investigated using X-ray diffraction (XRD). The resulting diffraction pattern provides crucial information regarding the phase composition and crystallographic properties of the material. Fig. 3 shows the XRD spectrum of fabricated CoSe@GO composite. Interpreting the provided XRD spectrum it is observed that a strong correlation between the experimental data and the characteristics of CoSe and GO is found. The prominent peaks in the spectrum (*e.g.*, at approximately 33.7°, 45.2°, 51.3°, 60.4°, and 62.8° 2*θ*) align well with the reference pattern for pure CoSe (JCPDS 89-2004), as indicated by the red and green colored vertical lines beneath the experimental data. This confirms the successful incorporation of the CoSe phase into the composite, a finding that is also consistent with the elemental analysis results discussed earlier. Furthermore, the presence of a broad, less intense peak centered around 2*θ* = 25° is clearly visible in the spectrum. This feature is attributed to the (002) facet graphene oxide (GO), indicating the presence of graphitic structures formed during the sample preparation. The broadness of this peak suggests the relatively disordered and few-layered nature of the GO. The sharp and well-defined peaks associated with CoSe suggest a good degree of crystallinity of the cobalt selenide phase within the composite.^20^ The indexing of specific peaks to the (101), (102), (110), (103), and (201) facets of CoSe, with the (101), (102), and (110) facets specifically pointing to the hexagonal close-packed structure, is supported by the peak positions observed in the spectrum. In comparison the GO peaks appeared at the ∼10° assigned to the (001) plane. This peak results from the introduction of epoxy, hydroxyl, and carboxyl oxygen-containing functional groups.

### 3.3 Raman analysis

**Fig. 4:**
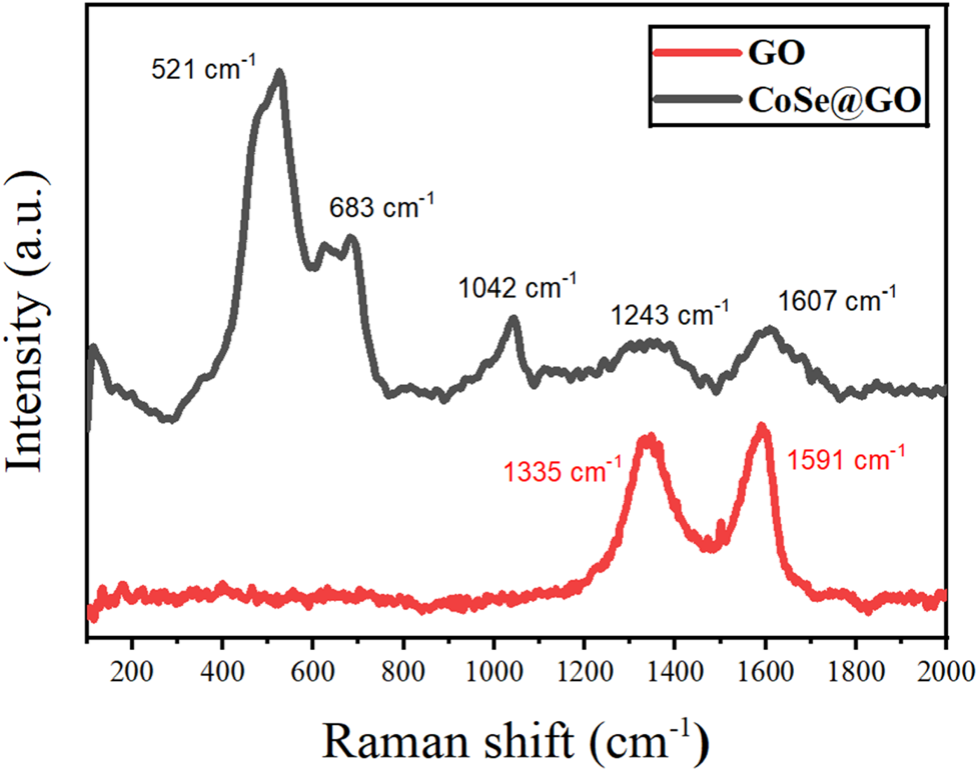
Raman spectra of GO and CoSe@GO nanocomposite.

The Raman spectroscopy was also used to elucidate the structural characteristics/vibration modes of hydrothermally synthesized nanocomposites (CoSe@GO) as shown in Fig. 4. The Raman spectrum of pristine GO displays the characteristic D band (1335 cm^−1^), associated with the breathing mode of sp^2^ carbon rings activated by structural defects and the G band (1591 cm^−1^) corresponding to the in-plane vibration of sp^2^ carbon atoms.^28,29^ The prominent D band confirms the high density of oxygen containing functional groups and structural disorder introduced during oxidation. Upon composite formation, the D and G bands persist, confirming graphene preservation, with a noticeable change in the *I*_D_/*I*_G_ ratio. This structural evolution elucidates strong interfacial interaction between CoSe nanoparticle and GO sheets. CoSe@GO exhibits pronounced Raman bands at ∼521 and 683 cm^−1^, corresponding to Co–Se lattice vibrational modes confirming crystalline cobalt selenide phase formation on the GO substrate. The intermediate band at ∼1042 cm^−1^ and broad features around 1243 cm^−1^ indicate defect-induced disorder and interfacial coupling between CoSe nanostructures and oxygenated functional groups of graphene oxide. This increased ratio suggests boosted defect density and partial reduction of GO during hydrothermal treatment resulting in smaller but various sp^2^ domains. In the spectrum, the slight shift and broadening of the G band suggest charge transfer between cobalt selenide and the carbon matrix, confirming strong electronic coupling within the hybrid structure. Moreover, the emergence of distinct Co–Se vibrational bands in the low-wavenumber range (200–700 cm^−1^) verifies the effective nucleation and crystallization of cobalt selenide on the GO surface, indicating true chemical bonding rather than simple physical mixing.^20^ Defect rich graphene domains facilitate rapid electron transfer, while cobalt selenide provides electroactive catalytic center that promote efficient redox reaction.

### 3.4 Electrochemical investigations for dopamine and uric acid

**Fig. 5:**
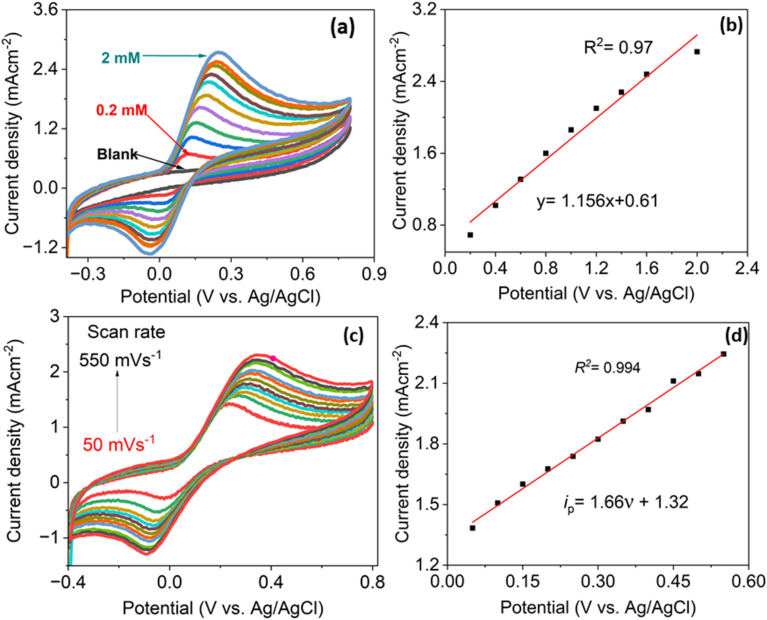
Cyclic voltammograms at (a) different concentrations of dopamine (0 to 2 mM) recorded at a fixed scan rate (c) at varying scan rates with a fixed dopamine concentration and (b and d) corresponding calibration curves.

The electrochemical performance of the CoSe-GO@GCE modified electrode was evaluated for the sensing of dopamine (DA) and uric acid (UA). This section details the observed electrochemical responses of the modified electrode to varying concentration and scan rate of DA and UA, highlighting its sensitivity, selectivity, and potential for simultaneous detection. All electrochemical measurements in this study were carried out exclusively in phosphate-buffer solution (PBS), which served as the supporting electrolyte for both characterization and sensing experiments. The PBS solution was prepared at pH 8.0, a mildly alkaline environment chosen to promote favorable oxidation kinetics for dopamine and uric acid by the electrochemical stability of the CoSe@GO composite. The buffer provides sufficient ionic strength and pH control enabling reliable charge transport and reproducible electrochemical responses during cyclic voltammetry, amperometry, and interference studies.^30^ Importantly, no external redox probe such as [Fe (CN_6_)]^3−^/^4−^ was used at any stage of electrochemical characterization. All reported currents, kinetic behaviors, and sensing performances arise solely from the intrinsic electrocatalytic activity of the CoSe@GO-modified electrode toward the target analytes in PBS. Fig. 5 shows the electrochemical response of DA at various concentrations at fixed scan rate and various scan rates at one fixed concentration of 2.0 mM DA. Based on the graph in Fig. 5(a) the electrochemical behavior of the CoSe-GO@GCE modified electrode surface in the presence of dopamine (DA) was thoroughly investigated by recording cyclic voltammograms across a range of DA concentrations, employing a constant scan rate to characterize the electrode's response dynamics. This approach allowed for a detailed evaluation of the redox processes of dopamine at the modified electrode interface. The *x*-axis represents the applied potential (V *vs.* Ag/AgCl), while the *y*-axis shows the measured current (µA). The observed increase in current with increasing DA concentration indicates a direct correlation between the analyte concentration and the electrochemical activity at the electrode surface. This suggests that the CoSe-GO@GCE electrode facilitates the oxidation and reduction of dopamine, leading to measurable current changes. The distinct peaks in the curves correspond to these oxidation and reduction processes of dopamine at the electrode surface. The rising peak currents with increasing DA concentrations demonstrate the electrode's sensitivity to dopamine, confirming its suitability for dopamine detection. This behavior is typical of electrochemical sensors, where the magnitude of the current signal is proportional to the analyte concentration. The CoSe-GO@GCE electrode shows promising performance for dopamine sensing due to its ability to transduce the chemical signal (dopamine concentration) into a measurable electrical signal (current). Fig. 5(b) presents the calibration curve of the CoSe-GO@GCE electrode for dopamine (DA) detection, illustrating a strong linear correlation between the anodic peak current density and the DA concentration. The high correlation coefficient (*R*^2^) of 0.97 confirms an excellent linear relationship over the investigated DA concentration range of 0.2 mM to 2 mM, consistent with the increasing current responses observed in the cyclic voltammograms of Fig. 5(a). The linear regression equation derived from the calibration curve is *y* = 1.15*x* + 0.60, where ‘*y’* represents the current density (mA cm^−2^) and ‘*x*’ represents the DA concentration (mM). The slope of this calibration curve, 1.15 mA cm^−2^ mM^−1^, signifies the sensitivity of the CoSe-GO@GCE electrode towards dopamine. A higher slope value indicates a greater change in current density for a given change in DA concentration, highlighting the electrode's ability to effectively transduce the dopamine concentration into an electrical signal. This sensitivity value underscores the potential of the CoSe-GO@GCE electrode for the quantitative determination of dopamine within this concentration range. The intercept of 0.60 mA cm^−2^ likely represents the background current density under the experimental conditions. The robust linear relationship and the calculated sensitivity demonstrate the suitability of the CoSe-GO@GCE electrode for reliable dopamine sensing applications. Fig. 5(c) displays cyclic voltammograms (CVs) of the CoSe-GO@GCE electrode in the presence of 2 mM dopamine (DA) at varying scan rates. These voltammograms were recorded to investigate the mass transfer phenomena governing the electrochemical reaction of DA at the electrode surface. Fig. 5(c) shows that as the scan rate increases from 50 to 550 mV s^−1^ in a 2 mM dopamine solution, both the oxidation and reduction peak currents of dopamine at the CoSe-GO@GCE electrode increase. This direct correlation between peak current and scan rate is a key characteristic of a diffusion-controlled process. The faster the potential is swept, the more dopamine is brought to the electrode surface, resulting in a higher reaction rate and thus larger peak currents for both the oxidation and reduction of dopamine. This indicates that the mass transfer of dopamine to the electrode surface is the limiting factor in the electrochemical reaction rate under these conditions. In graph 5(d) the linear relationship between peak current and the square root of scan rate further confirms the diffusion-controlled behavior of the redox process. This graph provides insights into the electrochemical behavior of the material under study, including its redox potential, reaction kinetics, and diffusion characteristics.

The high specificity of the CoSe@GO probe originates from its engineered CoSe-graphene heterointerface, where cobalt selenide provides abundant electroactive Co–Se redox sites and graphene ensures rapid charge transport. The active sites in CoSe are mainly the redox-active cobalt centers (Co^2+^/Co^3+^) particularly those exposed at edge and defect on the nanoparticle surface.^31^ These sites facilitate electron transfer and preferential adsorption of dopamine and uric acid, while selenium tunes the electronic structure of cobalt, enhancing catalytic activity and selectivity. As observed, the presence of dopamine and uric acid produces a pronounced, concentration-dependent oxidation response, indicating selective electrocatalysis at the CoSe@GO surface. The strong linearity (*R*^2^ = 0.97) and surface-controlled kinetics (*R*^2^ = 0.994) confirm that analyte oxidation proceeds *via* preferential adsorption and accelerated electron transfer at the CoSe@GO interface, rather than nonspecific diffusion-driven reactions. This synergistic probe architecture enables selective and reliable electrochemical profiling of dopamine and uric acid.

**Fig. 6:**
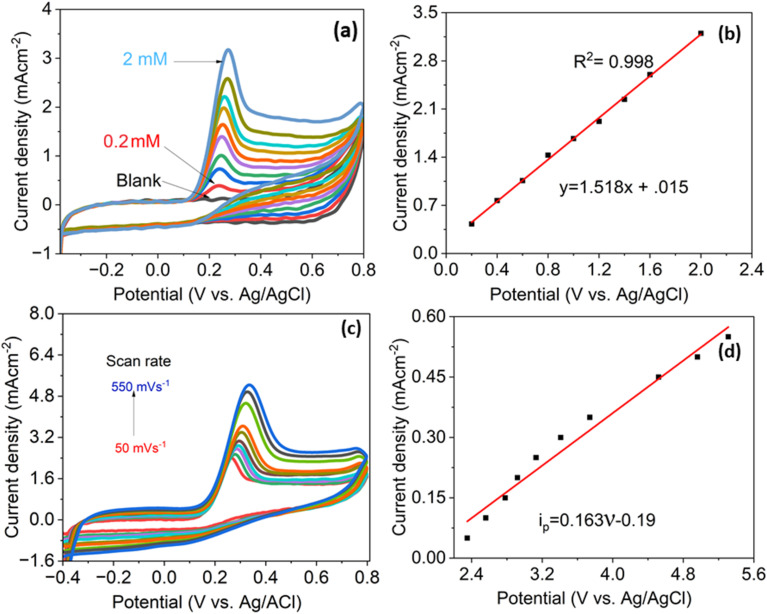
Cyclic voltammograms at (a) different concentrations of uric acid (0 to 2 mM) recorded at a fixed scan rate and (c) at varying scan rates with a fixed dopamine concentration and (b and d) corresponding calibration curves.

Fig. 6 shows the electrochemical response of UA at various concentrations at fixed scan rate and various scan rates at one fixed concentration of 2.0 mM UA is shown in Fig. 6. Based on the graph in Fig. 6(a) the cyclic voltammograms clearly illustrate the electrocatalytic performance of the CoSe@GO modified electrode for uric acid (UA) detection. While the blank electrolyte shows minimal faradaic activity, the introduction of UA results in a well-defined oxidation peak at approximately +0.33 V *vs.* Ag/AgCl, demonstrating the electrode's ability to facilitate UA oxidation at a relatively low potential. A key finding is the systematic increase in the oxidation peak current density with increasing UA concentrations (0.2 mM to 2.0 mM), indicating a strong correlation between the electrode response and analyte concentration, which is essential for quantitative sensing. The stability of the peak potential across different concentrations suggests a consistent reaction mechanism, while the absence of a corresponding reduction peak indicates an irreversible electrochemical oxidation process for UA on this modified surface. The enhanced performance is attributed to the synergistic effects of CoSe, providing the catalytic active sites, and GO, contributing high surface area and potentially enhancing electron transfer. Overall, the results strongly support the CoSe@GO composite as a promising material for developing sensitive electrochemical sensors for UA determination due to its clear concentration-dependent response and significant electrocatalytic activity. Fig. 6(b) displays the calibration curve for the CoSe@GO modified electrode, plotting peak current density against uric acid (UA) concentration. In the higher concentration range, approximately from 1.2 mM to 2.0 mM, the electrode response exhibits a distinct linear relationship described by the inset equation with a good coefficient of determination (*R*^2^ = 0.94). The sensitivity value as of 1.51 mA cm^−2^ quantifies the change in current density signal per unit change in UA concentration, indicating that for every 1 mM increase in UA concentration between 1.2 mM and 2.0 mM, the measured current density increases. The reasonably high *R*^2^ value associated with this slope confirms the reliable and proportional response of the electrode in this higher concentration range, suitable for quantitative measurements.

**Fig. 7:**
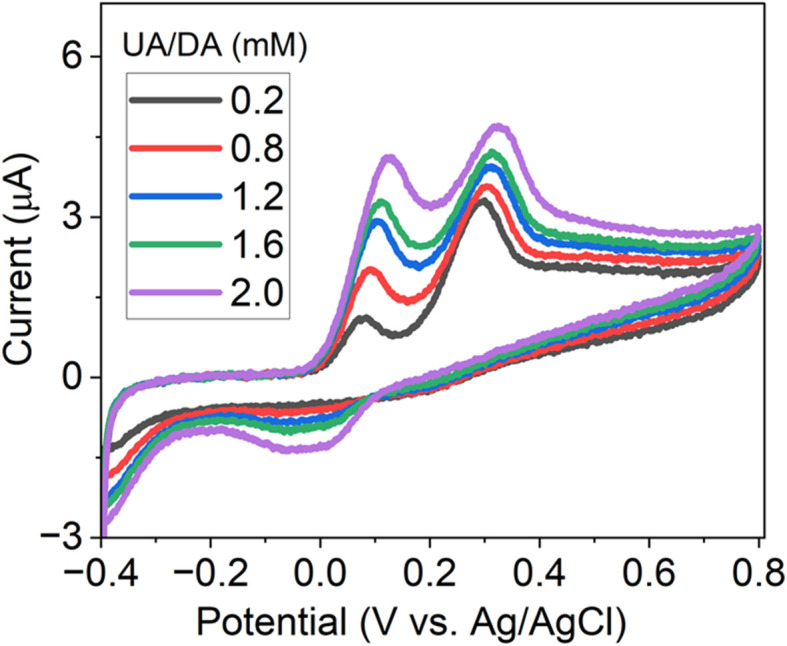
Cyclic voltammograms for simultaneous electrochemical sensing of DA and UA with equimolar concentrations.

To evaluate the practical applicability and selectivity of the CoSe@GO modified electrode, its electrochemical response was investigated in the simultaneous presence of two biologically relevant analytes, uric acid (UA) and dopamine (DA). The resulting cyclic voltammograms, recorded in solutions containing simultaneously increasing concentrations of both analytes, are presented in the Fig. 7. This cyclic voltammogram demonstrates the simultaneous electrochemical detection of uric acid (UA) and dopamine (DA) using the CoSe@GO modified electrode. The curves were recorded in solutions containing equal and increasing concentrations of both UA and DA, ranging from 0.2 mM to 1.2 mM each. Notably, two distinct and well-separated anodic peaks are observed within the potential window scanned (−0.4 V to +0.8 V *vs.* Ag/AgCl). The first oxidation peak, appearing at approximately +0.18 V, corresponds to the electrochemical oxidation of dopamine (DA), while the second peak, centered around +0.33 V, is attributed to the oxidation of uric acid (UA). The clear potential difference (around 150 mV) between these two peaks is significant, as it indicates the electrode's ability to effectively differentiate between the two analytes without substantial signal interference or overlap. Furthermore, as the concentrations of both UA and DA are simultaneously increased, the current densities of both respective peaks show a clear and progressive increase. This concentration-dependent response for both species simultaneously underscores the CoSe@GO electrode's capability for quantitative, selective multi-analyte sensing, highlighting its promising electrocatalytic activity towards both dopamine and uric acid.

**Fig. 8:**
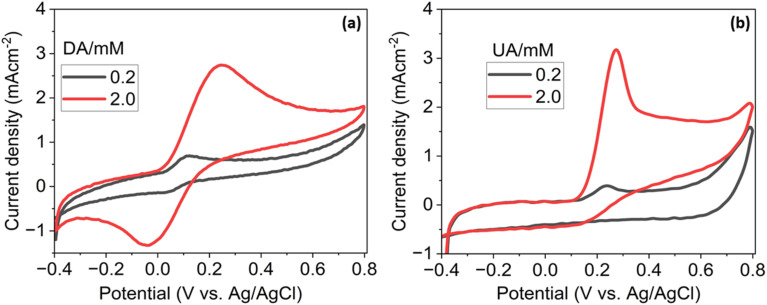
Comparison *via* cyclic voltammograms for the electrochemical detection of (a) dopamine and (b) uric acid at 0.2 mM and 2.0 mM using the CoSe@GO modified electrode.

To better understand the basis for selective detection, the individual electrochemical responses of dopamine (DA) and uric acid (UA) were examined separately on the CoSe@GO modified electrode. The Fig. 8 show the cyclic voltammograms obtained for each analyte at two representative concentrations, 0.2 mM and 2.0 mM. These cyclic voltammograms compare the individual electrochemical responses of dopamine (DA) and uric acid (UA) on the CoSe@GO electrode at 0.2 mM and 2.0 mM concentrations. Dopamine (DA) shows quasi-reversible redox behavior with an oxidation peak around +0.2 to +0.25 V and a reduction peak near −0.05 V (Fig. 8(a)). The peak currents significantly increase with concentration. Uric Acid (UA) exhibits an irreversible oxidation process with a distinct peak consistently around +0.33 V (Fig. 8(b)). No reduction peak is observed. The oxidation peak current clearly increases with concentration. The CoSe@GO electrode oxidizes DA at a lower potential than UA (∼+0.2–0.25 V *vs.* +0.33 V). Furthermore, DA's electrochemistry is quasi-reversible, while UA's is irreversible. This distinct difference in oxidation potential and reversibility confirms the electrode's ability to differentiate between the two analytes.

### 3.5 Amperometric response

**Fig. 9:**
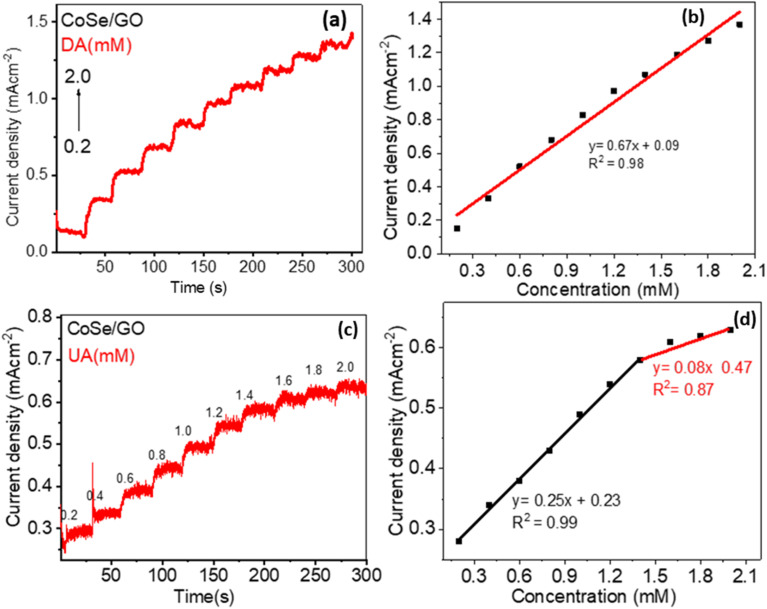
Amperometric response for varying concentrations of (a) dopamine and (c) uric acid and (b and d) related calibration curves.

To further evaluate the analytical performance and real-time sensing capability of the CoSe@GO modified electrode for dopamine (DA) and uric acid detection, amperometric measurements were performed under optimized conditions. The Fig. 9(a) showcases the amperometric performance of the CoSe@GO modified electrode for detecting dopamine (DA). The plot displays the current density response over time as successive additions of DA are made to the electrolyte, increasing the concentration stepwise from an initial level up to approximately 2.0 mM (starting likely near 0.2 mM based on annotations, though the exact increments aren't specified for each step). The resulting staircase pattern is characteristic of this technique and clearly demonstrates the sensor's effective response to the analyte. With each addition of DA, there is a rapid increase in the oxidation current density, which quickly reaches a stable steady-state plateau before the next addition. This signifies the sensor's fast response time and its ability to reliably detect changes in DA concentration. The height of each step, representing the change in current density for a given concentration increment, reflects the sensor's sensitivity at the applied potential. The well-defined and reproducible steps confirm the suitability of the CoSe@GO electrode for the quantitative amperometric determination of dopamine.^32^

This graph presents the calibration curve constructed from the previous amperometric measurements plotting the steady-state current density values against the corresponding dopamine (DA) concentrations. A clear linear relationship is observed across the tested concentration range (approx. 0.2 mM to 2.0 mM). The data is well-fitted by the linear regression equation *y* = 0.67*x* + 0.09, yielding an excellent coefficient of determination (*R*^2^ = 0.98). This high *R*^2^ value confirms the strong proportionality between the amperometric current response and the DA concentration, validating the suitability of the CoSe@GO electrode for quantitative analysis using this technique. Furthermore, based on analysis accounting for signal-to-noise ratios at lower concentrations the limit of detection (LOD) for DA was determined to be 1.59 mM. Taken together, the excellent linearity demonstrated by the high *R*^2^ value, the defined sensitivity, the calculated LOD, and the stable steady-state currents observed previously highlight the reliable and promising performance characteristics of the CoSe@GO sensor for amperometric dopamine detection. Fig. 9(c) illustrates the real-time amperometric performance of the CoSe@GO modified electrode for the detection of uric acid (UA) at a fixed applied potential. The graph plots the current density response (mA cm^−2^) against time (s) during the successive, stepwise addition of UA into the electrochemical cell. The concentrations corresponding to each step, ranging from 0.2 mM to 2.0 mM, are indicated on the graph. A characteristic staircase profile is clearly observed, where each incremental addition of UA results in a distinct and rapid increase in the oxidation current density. Importantly, the current quickly reaches a stable steady-state plateau after each addition, indicating a fast response time and efficient electro catalysis at the electrode surface. The well-defined and reproducible nature of these current steps demonstrates a clear dependence of the sensor's signal on the UA concentration, highlighting the suitability of the CoSe@GO electrode for sensitive and quantitative amperometric determination of uric acid. Fig. 9(d) displays the calibration curve obtained from the amperometric detection of uric acid (UA), plotting the steady-state current density against the corresponding UA concentration.

The plot clearly shows two distinct linear regions. In the primary working range, from approximately 0.2 mM up to 1.4 mM, the sensor demonstrates excellent linearity, as described by the equation *y* = 0.25*x* + 0.23 and validated by a high coefficient of determination (*R*^2^ = 0.99). This high degree of linearity ensures that the sensor provides a highly predictable and proportional response to changes in UA concentration, enabling reliable and accurate quantification within this important physiological range. The complete set of electrochemical parameters derived from chrono analysis of dopamine a neurotransmitter and uric acid is listed in Table S1.

The applied amperometric potentials of 0.2 V for dopamine and 0.3 V for uric acid were selected based on preliminary cyclic voltammetry (CV) studies performed at the CoSe@GO probed electrode. The CV profiles showed well-defined and distinct oxidation peaks for DA and UA centered around 0.2 V and 0.3 V indicating their lowest overpotential oxidation at these potentials. Amperometric measurements were therefore conducted at these values to ensure maximum faradaic current response while minimizing background current and interference from other electroactive species. The difference in optimal potentials arises from the intrinsic oxidation kinetics and adsorption strengths of DA and UA on the Co–Se active sites, with DA undergoing faster electron transfer due to stronger π–π interactions with graphene, whereas UA requires a slightly higher potential because of its more complex oxidation pathway.^33^ Thus, selecting 0.2 V and 0.3 V ensures high sensitivity and improved selectivity in the amperometric response for each analyte.

**Fig. 10:**
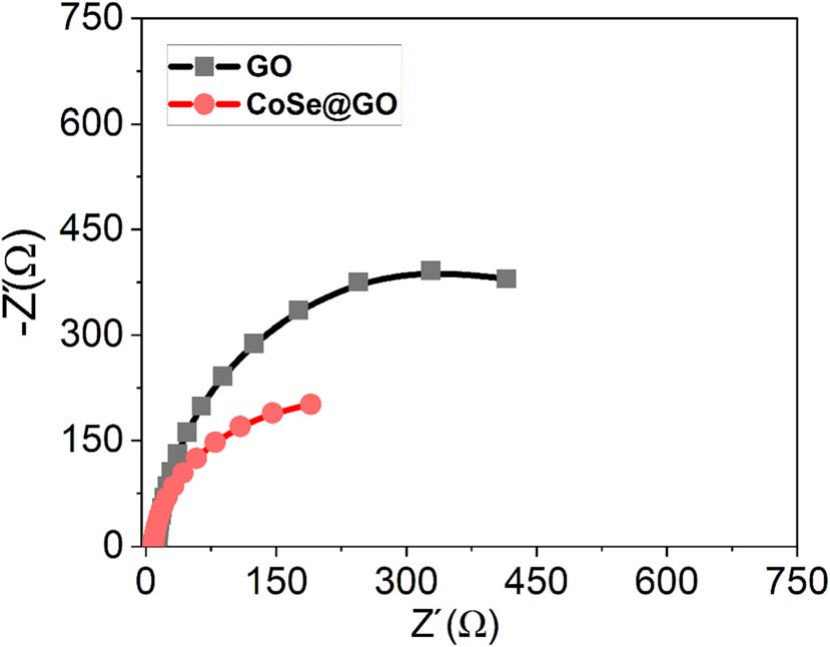
Comparative EIS responses reflecting charge transfer resistance and redox activeness.

The Nyquist plots obtained from electrochemical impedance spectroscopy (EIS) provide clear evidence for the enhanced charge-transfer characteristics of the CoSe@GO composite compared with its individual components (Fig. 10). In EIS, the diameter of the high-to-medium frequency semicircle corresponds to the charge-transfer resistance (*R*_ct_) at the electrode–electrolyte interface. As shown, GO exhibits a relatively large semicircle, reflecting its intrinsically poor electrical conductivity and sluggish interfacial electron transport. In contrast, CoSe@GO displays a very small semicircle than GO due to its higher intrinsic conductivity and redox-active nature indicating a significantly lower *R*_ct_ and faster charge-transfer kinetics. This improvement arises from the synergistic integration of CoSe with the conductive GO network, where GO acts as an electron highway that facilitates rapid electron transport while simultaneously preventing CoSe aggregation and increasing the number of accessible electroactive sites.^34^ Consequently, the reduced interfacial resistance and improved electronic flow with CoSe@GO underpins its superior electrochemical performance relative to pure GO demonstrating the effectiveness of composite engineering for enhanced charge-transfer behavior.

**Fig. 11:**
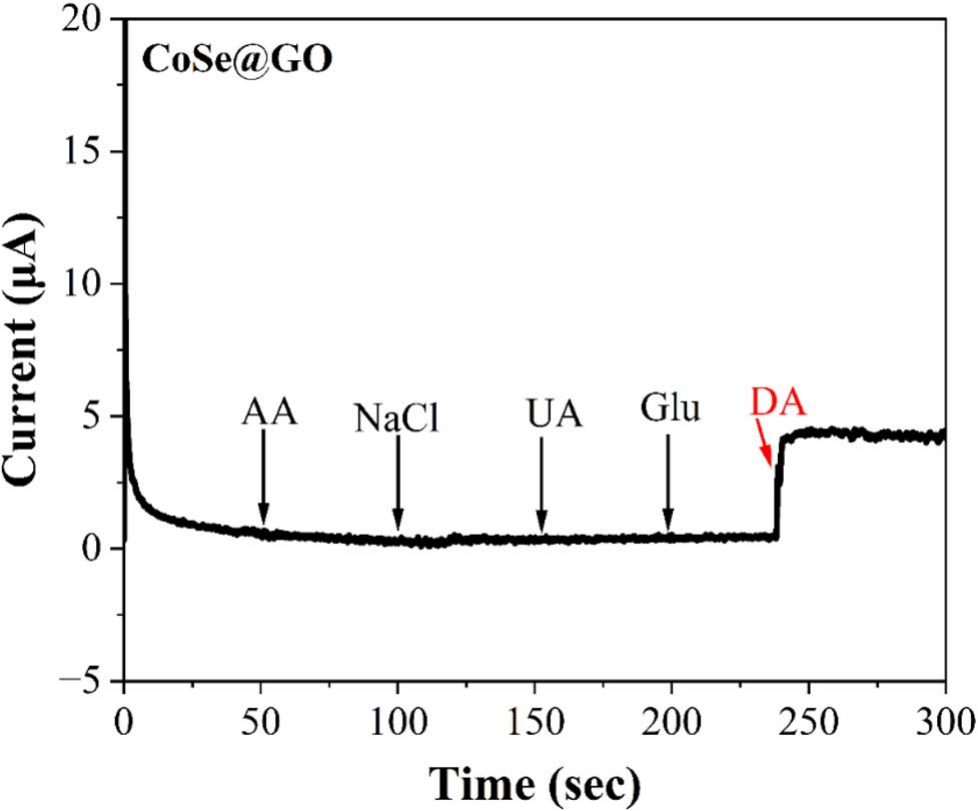
Effect of interference exhibiting selectivity against common interferents.

The amperometric interference response highlights the role of the CoSe@GO electrode material in achieving high selectivity toward dopamine as shown in Fig. 11. As observed, the addition of common interfering species such as ascorbic acid (AA), NaCl, uric acid (UA), and glucose (Glu) induces negligible current changes, whereas DA injection results in a pronounced and rapid current increase. This behavior originates from the intrinsic properties of the electrode material, Co–Se provides abundant electroactive sites and strong electrocatalytic activity that lower the activation energy for DA oxidation, while the graphene oxide (GO) matrix offers a high surface area, conductive pathways, and oxygen-containing functional groups that facilitate electron transport during DA oxidation.^35^ Consequently, the rationally engineered electrode material governs interfacial charge transfer, adsorption selectivity, and reaction kinetics, leading to excellent anti-interference capability and reliable DA sensing performance.

The comparative cyclic voltammetry responses of GO@GCE and CoSe-modified electrodes (without GO) illustrate the distinct electrochemical roles of GO and CoSe, providing a mechanistic basis for the superior performance of the CoSe than GO alone. As shown in Fig. S1(a), GO@GCE exhibits relatively low anodic currents and broadened redox features for dopamine (DA), reflecting its limited intrinsic electrocatalytic activity. In contrast, CoSe (Fig. S1(b)) delivers significantly higher oxidation currents, confirming the strong catalytic activity of CoSe towards DA oxidation.

However, the response is dominated by a steep current rise at higher potentials, indicative of less controlled interfacial kinetics and limited peak definition. The CoSe active sites introduces efficient electron-conduction pathways resulting in enhanced peak currents, improved peak separation for DA and UA oxidation.^36^ The scan-rate-dependent CVs (Fig. S1(c) and (d)) further reveal that GO shows quasi-reversible behavior with moderate current scaling, while CoSe displays faster kinetics. The similar trend is observed for UA detection with the same comparison respectively (Fig. S2(a)–(d)).

**Scheme 2:**
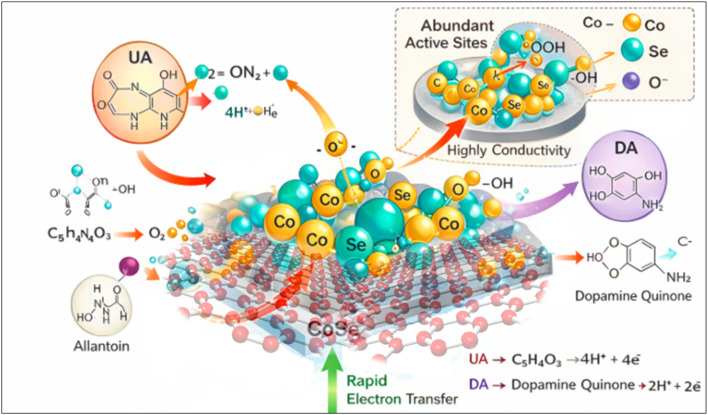
Catalysis mechanism of CoSe@GO in detecting uric acid and dopamine.

The electrocatalytic sensing mechanism of CoSe@GO towards uric acid (UA) and dopamine (DA) originates from the synergistic coupling between cobalt selenide (CoSe) nanocrystals and the conductive graphene oxide (GO), which together regulate adsorption, charge transfer, and reaction kinetics at the electrode–electrolyte interface. Structurally, CoSe provides abundant exposed Co–Se active sites with mixed-valence cobalt centers, while GO offers high-surface area and π-conjugation that promotes fast electron transport and prevents CoSe agglomeration as given in Scheme 2.^37^

For uric acid detection, UA molecules are preferentially adsorbed onto the CoSe@GO surface through a combination of hydrogen bonding with functional groups of GO and electrostatic interactions with Co centers.^38^ Upon adsorption, UA undergoes an irreversible electro-oxidation involving a four electron–proton transfer to yield allantoin as the final oxidation product. The Co–Se lattice facilitates this process by lowering the activation energy for UA oxidation, while GO efficiently extracts the generated electrons and transports them to the electrode. In contrast, dopamine sensing proceeds *via* a quasi-reversible redox mechanism. DA molecules are strongly enriched at the CoSe@GO interface due to π–π interactions between the aromatic ring of dopamine and the sp^2^ carbon domains of GO.^39^ At the catalytic CoSe sites, DA is oxidized to dopamine quinone through a two electron–proton process. CoSe accelerates the electron exchange kinetics, while GO ensures fast lateral electron conduction and minimizes surface fouling by oxidation products. Crucially, the distinct oxidation potentials of DA (0.2–0.25 V) and UA (0.33 V) arise from their different adsorption configurations and reaction energetics on the CoSe@GO surface enabling selective detection. Overall, the sensing performance of CoSe@GO is governed by a dual mechanism, where CoSe acts as the primary electrocatalyst providing active redox sites, and GO serves as an electron-conducting and adsorption-regulating matrix. A comparison table (Table 1) benchmarks the CoSe@GO sensor against recent DA and UA sensors, showing its good sensitivity. Unlike many reported sensors that trade sensitivity for narrow ranges or poor selectivity, CoSe@GO offers a balanced performance.

**Table 1:** A comparative table with electrochemical biosensing parameters

Electrode material	Analyte	Sensitivity (µA mM^−1^)	LOD (mM)	Ref.
MoSe@GCE	DA/UA	—	0.005/0.001	40
FeSe_2_@rGO	UA	820	0.002	41
3D rGO Ti_3_C_2_	DA, UA	900	0.06/0.08	42
PdNPs/rGO^43^	DA, UA	—	0.000184/0.0016	42
GR/MWCNT/GCE	DA/UA	1.38/0.181	0.58/7.30	44
ErGO/PEDOT:PSS	DA/UA	—	0.0005/0.0004	45
MoSe_2_/rGO	UA		0.0284 µM	46
CoSe@GO	DA/UA	DA-1150	DA-1.59	This work
		UA-1500	UA-0.33	

## 4 Conclusions

CoSe@GO nanoparticles were effectively prepared through a facile hydrothermal method and applied as an effective electrocatalyst for sensing crucial biomolecules. Electrochemical evaluations, employing cyclic voltammetry and chronoamperometry, demonstrated the enhanced performance of the CoSe@GO modified GCE towards the detection of both dopamine (DA) and uric acid (UA). The developed sensor exhibited a favorable linear response for both analytes within the concentration range of 0.2–2.0 mM. Notably, the sensor achieved low detection limits of 1.59 mM for dopamine and 0.33 mM for uric acid, respectively. Furthermore, the CoSe@GO composite material displayed pretty good selectivity, enabling the distinct detection of these analytes, along with commendable stability. These promising results highlight the significant potential of the fabricated CoSe@GO electrode material for developing advanced electrochemical sensors, potentially paving the way for its application in flexible and wearable devices for practical biosensing applications.

## Author contributions

Munira Khalid, Musarrat Younas: conceptualization, experiments, writing. Abid Ali, Amel Y. Ahmed, Imene Bayach: supervision, drafting, proof reading, funding acquisition. Arif Nazir, Warda Mansur: formal analysis, validation. Murat Kaleli, Salih Akyürekli: material characterization.

## Conflicts of interest

The authors declare that they have no known competing financial interests or personal relationships that could have appeared to influence the work reported in this paper.

## Funding

This work was financially supported by the Deanship of Scientific Research at King Faisal University, Saudi Arabia under Ambient Researcher [Grant KFU263018].

## Supplementary Material

RA-OLF-D5RA08825A-s001

## Data Availability

Data will be available on reasonable request. Supplementary information (SI): electrochemical analysis. See DOI: https://doi.org/10.1039/d5ra08825a.
