# Supplementary material for: Hydrothermally synthesized cobalt selenide graphene nanocomposite as a sensitive probe for electrochemical profiling of dopamine and uric acid
Source: RSC Adv. 2026 Jul 2;16(34):32175–88. doi: 10.1039/d5ra08825a (PMC13326228; doi:10.1039/d5ra08825a)
Supplement: RA-016-D5RA08825A-s001 [file RA-016-D5RA08825A-s001.pdf]

Supplementary Information (SI) for RSC Advances.
This journal is © The Royal Society of Chemistry 2026

Hydrothermally Synthesized Cobalt Selenide@Graphene Nanocomposite as Sensitive Probe for Electrochemical Profiling of Dopamine and Uric Acid

Munira Khalid[1], Musarrat Younas[1], Abid Ali[2]*, Arif Nazir[2], Amel Y. Ahmed[3]*, Imene Bayach[3]*, Warda Mansur[2], Murat Kaleli[4], Salih Akyürekli[4]

[1]Department of Chemistry Women University of Azad Jammu & Kashmir Bagh 12500, Pakistan

[2]Department of Chemistry, The University of Lahore, 1-Km Defence Road, Lahore, Pakistan

[3]Department of Chemistry, Faculty of Science, King Faisal University, Al Ahsa 31982, Saudi Arabia

[4]Suleyman Demirel University, Innovative Technologies Application and Research Center, 32260 West Campus Çünür, Isparta, Turkey

Corresponding Author

***Dr. Abid Ali**

Email: abid.ali@chem.uol.edu.pk

Department of Chemistry, The University of Lahore, 1-Km Defence Road, Lahore 54590, Pakistan

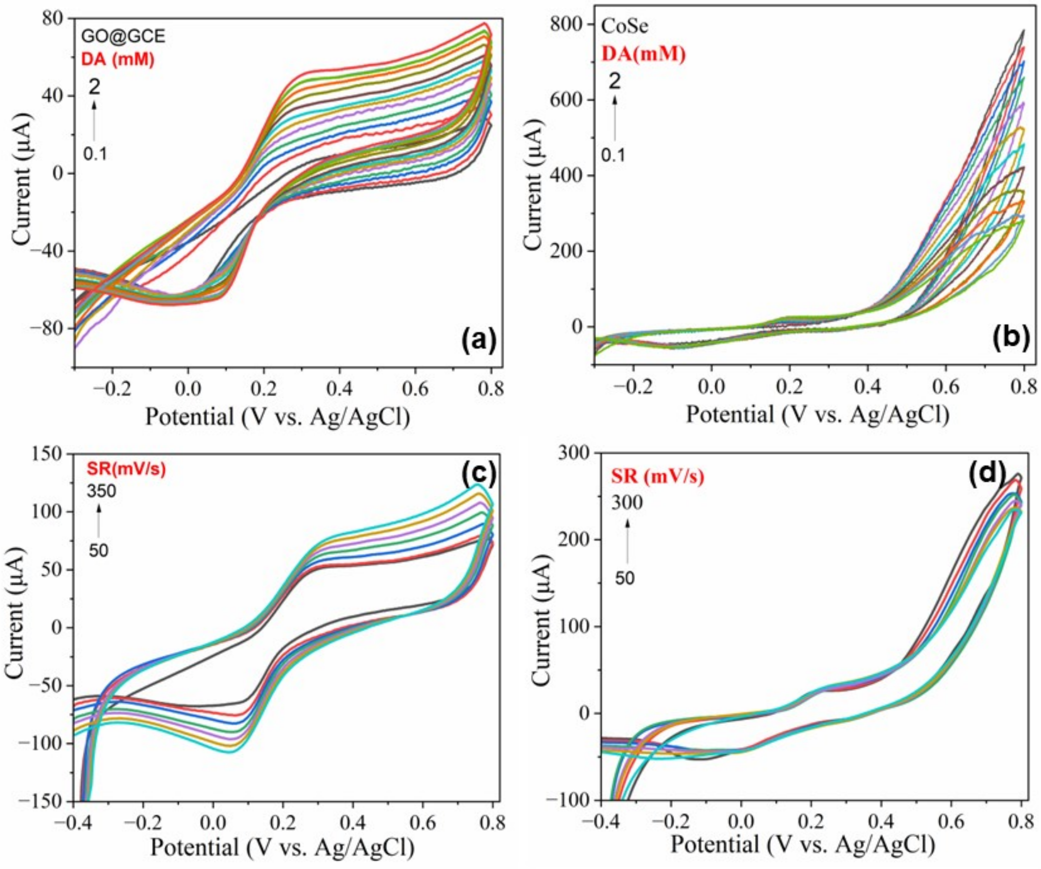

Figure S1. Comparative CV analysis in the electrochemical detection of dopamine towards **(a)** GO **(b)** CoSe **(c-d)** The corresponding Scan rate curves.

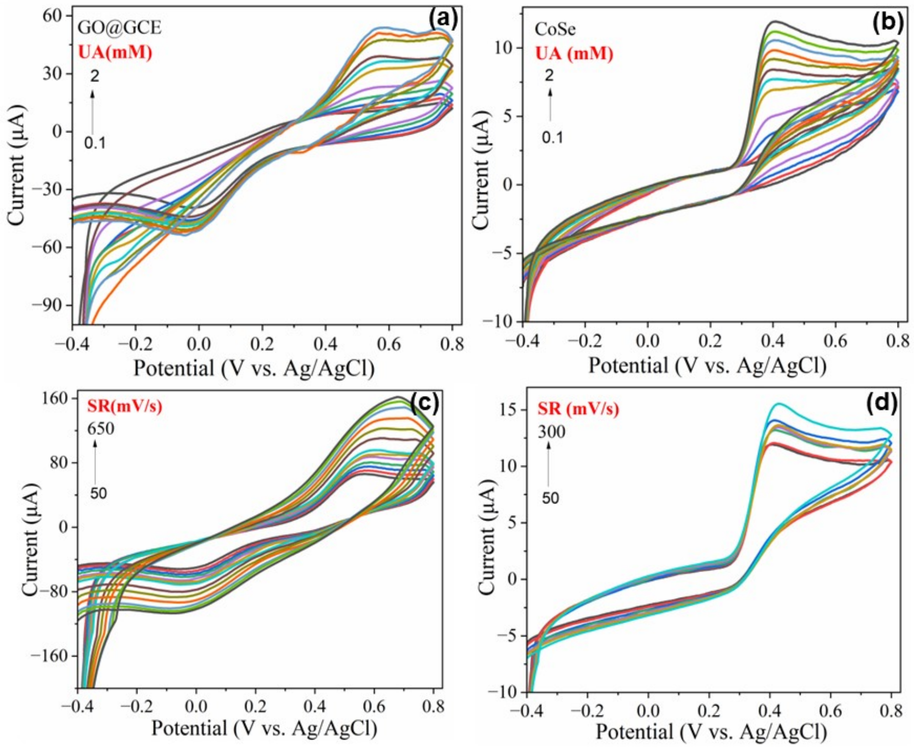

Figure S2. Comparative CV analysis in the electrochemical detection of Uric acid towards **(a)** GO **(b)** CoSe **(c-d)** The corresponding Scan rate curves.

Table S1. Electrochemical parameters for the sensing of dopamine and uric acid via chrono.

Analyte	Sensitivity ($mAcm^{-2}mM^{-1}$)	Linear Range (mM)
**Dopamine**	0.67	0.2-2.0
**Uric Acid**	0.25	0.2-1.2<br>1.2-2.0
